# Randomised controlled trial of Alexander technique lessons, exercise, and massage (ATEAM) for chronic and recurrent back pain

**DOI:** 10.1136/bmj.a884

**Published:** 2008-08-19

**Authors:** Paul Little, George Lewith, Fran Webley, Maggie Evans, Angela Beattie, Karen Middleton, Jane Barnett, Kathleen Ballard, Frances Oxford, Peter Smith, Lucy Yardley, Sandra Hollinghurst, Debbie Sharp

**Affiliations:** 1 Primary Care Group, Community Clinical Sciences Division, University of Southampton, Aldermoor Health Centre, Southampton SO16 5ST; 2 School of Psychology, University of Southampton; 3 Department of Social Statistics, University of Southampton; 4 Academic Unit of Primary Health Care, Department of Community Based Medicine, University of Bristol; 5 Society of Teachers of the Alexander Technique, London

## Abstract

**Objective** To determine the effectiveness of lessons in the Alexander technique, massage therapy, and advice from a doctor to take exercise (exercise prescription) along with nurse delivered behavioural counselling for patients with chronic or recurrent back pain.

**Design** Factorial randomised trial.

**Setting** 64 general practices in England.

**Participants** 579 patients with chronic or recurrent low back pain; 144 were randomised to normal care, 147 to massage, 144 to six Alexander technique lessons, and 144 to 24 Alexander technique lessons; half of each of these groups were randomised to exercise prescription.

**Interventions** Normal care (control), six sessions of massage, six or 24 lessons on the Alexander technique, and prescription for exercise from a doctor with nurse delivered behavioural counselling.

**Main outcome measures** Roland Morris disability score (number of activities impaired by pain) and number of days in pain.

**Results** Exercise and lessons in the Alexander technique, but not massage, remained effective at one year (compared with control Roland disability score 8.1: massage -0.58, 95% confidence interval -1.94 to 0.77, six lessons -1.40, -2.77 to -0.03, 24 lessons -3.4, -4.76 to -2.03, and exercise -1.29, -2.25 to -0.34). Exercise after six lessons achieved 72% of the effect of 24 lessons alone (Roland disability score -2.98 and -4.14, respectively). Number of days with back pain in the past four weeks was lower after lessons (compared with control median 21 days: 24 lessons -18, six lessons -10, massage -7) and quality of life improved significantly. No significant harms were reported.

**Conclusions** One to one lessons in the Alexander technique from registered teachers have long term benefits for patients with chronic back pain. Six lessons followed by exercise prescription were nearly as effective as 24 lessons.

**Trial registration** National Research Register N0028108728.

## Introduction

Back pain is a common condition managed in primary care and one of the commonest causes of disability in Western societies.^1^ ^2^ As yet few interventions have been proved to substantially help patients with chronic back pain in the longer term.

Supervised exercise classes—mainly strengthening and stabilising exercises—probably have moderate benefit for chronic pain.^3^ ^4^ ^5^ ^6^ ^7^ A trial of advice from a doctor to take aerobic exercise showed short term benefit for acute pain,^8^ but the evidence of longer term benefit for chronic or recurrent pain and for exercise “prescriptions” is lacking.^9^

Lessons in the Alexander technique offer an individualised approach designed to develop lifelong skills for self care that help people recognise, understand, and avoid poor habits affecting postural tone and neuromuscular coordination. Lessons involve continuous personalised assessment of the individual patterns of habitual musculoskeletal use when stationary and in movement; paying particular attention to release of unwanted head, neck, and spinal muscle tension, guided by verbal instruction and hand contact, allowing decompression of the spine; help and feedback from hand contact and verbal instruction to improve musculoskeletal use when stationary and in movement; and spending time between lessons practising and applying the technique (also see appendix on bmj.com).

The Alexander technique is thus distinct from manipulation,^10^ back schools,^11^ and conventional physiotherapy.^12^ The practice and theory of the technique, in conjunction with preliminary findings of changes in postural tone and its dynamic adaptability to changes in load and position,^13^ ^14^ ^15^ support the hypothesis that the technique could potentially reduce back pain by limiting muscle spasm, strengthening postural muscles, improving coordination and flexibility, and decompressing the spine. A small trial, not fully reported, showed promising short term results for back pain.^16^ We are not aware of a trial reporting long term results.

Systematic reviews and a recent trial highlighted the importance of research to assess the effectiveness of holistic therapeutic massage^17^ ^18^ ^19^; we particularly wanted to assess massage as it provides no long term educational element, in contrast with lessons in the Alexander technique.

We determined the effectiveness of six or 24 lessons in the Alexander technique, massage therapy, and advice from a doctor to take exercise (using an exercise prescription) with nurse delivered behavioural counselling for patients with chronic or recurrent back pain.

## Methods

We recruited 64 general practices in the south and west of England in two centres (Southampton and Bristol) on the basis of geographical availability of teachers of the Alexander technique and massage therapists; 152 teachers and therapists agreed to participate. Each practice wrote to a random selection of patients who had attended with back pain in the past five years (see box for inclusion criteria, mostly similar to the United Kingdom back pain exercise and manipulation trial^7^ for comparability). Patients were given information that there was suggestive preliminary evidence to support each intervention (Alexander technique, massage, and exercise). We recruited patients from 8 July 2002 to 22 July 2004.

**Inclusion and exclusion criteria of patients with back pain in past five years**

**Inclusion criteria: to identify those with significant recurrent pain or chronic pain**

- Presentation in primary care with low back pain more than three months previously (to exclude first episodes)
- Currently scoring 4 or more on the Roland disability scale
- Current pain for three or more weeks (to exclude recurrence of short duration)

**Exclusion criteria**

- Previous experience of Alexander technique
- Patients under 18 and over 65 (serious spinal disease more likely)
- Clinical indicators of serious spinal disease^20^
- Current nerve root pain (below knee in dermatomal distribution), previous spinal surgery, pending litigation (outcome may be different, groups too small to analyse)
- History of psychosis or major alcohol misuse (difficulty completing outcomes)
- Perceived inability to walk 100 m (exercise difficult)

### Randomisation

At the baseline appointment, after informed written consent had been obtained, participants were randomised to one of eight groups by the practice nurse telephoning the central coordinating centre in Southampton (table 1 and appendix on bmj.com). A statistician had prepared a secure program using computer generated random numbers so that the next allocation could not be guessed. For each practice contributing 10 patients a block of eight numbers existed, and two were added from a block that supplied four other practices. Practices were not told how many patients would be recruited to each trial group or informed of the block randomisation. When possible each practice was matched to two Alexander technique teachers.

**Table 1 tbl1:** Trial groups for patients with chronic or recurrent back pain

Intervention	No exercise	Exercise*
Normal care	Group 1 (control)	Group 5
Therapeutic massage (6 sessions)†	Group 2	Group 6
Alexander technique lessons (n=6)‡	Group 3	Group 7
Alexander technique lessons (n=24)§	Group 4	Group 8

*Doctor prescription and up to three sessions of behavioural counselling with practice nurse. Doctor exercise prescription was scheduled six weeks into trial to allow groups 7 and 8 to have some Alexander technique lessons before starting exercise but not to delay any further the start for group 5.

†One session a week for six weeks.

‡Two lessons a week for two weeks then one lesson a week for two weeks.

§Twenty two lessons over five months, initially two a week for six weeks, one a week for six weeks, one fortnightly for eight weeks, and one revision lesson at seven months and one at nine months.

### Outcome measures

The first primary outcome measure was disability, measured using the Roland Morris disability questionnaire. Patients indicate the number of specified activities or functions limited by back pain^21^ ^22^ (for example, getting out of the house less often, walking more slowly than usual, not doing usual jobs around the house). The scale is designed for self report and has good validation characteristics.^23^ The second primary outcome measure was number of days in pain during the past four weeks^24^ (a four week period facilitated recall): this is distinct from intensity of pain or disability.^24^ ^25^

Secondary outcome measures were quality of life, measured using the short form 36,^26^ and secondary measures for back pain^21^: pain and disability using the Von Korff scale^24^ and Deyo “troublesomeness” scale,^21^ overall improvement using health transition,^23^ and fear avoidance beliefs for physical activity.^27^

For other measures we asked patients to agree or disagree with statements on 7 point scales from 0=strongly agree to 7=strongly disagree. We developed a back health scale (my health has improved, I feel better, I have less back pain, I am able to be more active; Cronbach’s a=0.96), and a modified enablement instrument^28^ (mean of six items: I am able to cope better with life, I am able to understand my (back) problem better, I am able to cope better with my (back) problem, I am better able to keep myself healthy, I am more confident, I am able to help myself; Cronbach’s a=0.96).

We measured outcomes at baseline, three months, and one year using postal questionnaires, with two mailings to non-responders and telephone follow-up for a smaller dataset (Roland disability scale, days in pain, Von Korff scale, health transition) for those not responding. Data entry was blind to study group.

### Sample size

The sample size was calculated using the Nquery program. The Medical Research Council back pain working group for the back pain exercise and manipulation trial^7^ agreed that a 2.5 point change on the Roland disability scale was a clinically important change in the context of several sessions of manipulation (that is, a relatively intensive intervention^29^). In the context of both intensive and less intensive interventions we assumed that changes in the range 1.5 to 2.5 could therefore be important. This was also justified in our cohort: patients who rated their back pain as slightly improved after one year compared with those rating their pain as not improved (a difference of 1 point on a 7 point scale) had changed Roland disability scores by an additional 2.2 points; 50% of patients achieving this change (a 1.1 point difference) might still be important clinically. We assumed the standard deviation to be 4.^7^ ^30^ The limiting element in the sample size calculations was the Alexander technique factor. For a=0.01 and 80% power^31^ and assuming the interventions could achieve an effect in the clinically important range (six Alexander technique lessons 1.5 points lower than normal care, massage 2 points lower, and 24 Alexander technique lessons 2.5 points lower) then 292 patients were required for the Alexander technique factor (73 in each group), or 365 allowing for 20% loss to follow-up. The trial had no cluster design effects as it was individually randomised. We wanted, however, to allow for clustering effects (of practice, general practitioner, and teacher or therapist) if these proved statistically significant: we included an inflation factor of 1.45, which required 529 patients (365×1.45), or 536 in total to provide eight balanced factorial groups.

### Analysis

The analysis plan was agreed in advance by the trial management group. The primary analysis was an analysis of covariance for a factorial study at one year for the primary outcome between groups (Roland disability score) and for the secondary outcomes. The days in pain data were skewed so we used non-parametric (quantile) regression. We assessed interaction between factors before reporting the main effects: those of the Alexander technique factor are reported controlling for the effect of exercise and those of the effect of exercise are reported controlling for the Alexander technique factor. As the study was powered for only moderately large interactions we also report the individual groups for the main outcomes at one year. We assessed the statistical significance of clustering by therapist, teacher, and practice, and if these were not significant we did not allow for clustering in the models.

## Results

Most eligible patients who responded agreed to attend for assessment (figure[Fig fig1]). We wrote to 687 consecutive patients who did not respond to the original invitation, to assess potential eligibility of non-responders: 553 responded, of whom only six were eligible. A total of 579 people were randomised and completed the baseline questionnaires, 469 (81%) completed the questionnaires at three months, and 463 (80%) the questionnaires at 12 months. Responders at one year were more likely to have left full time education later and to be self employed or homemakers; response was not related to baseline Roland disability scores. Including education and employment status in the final analysis did not alter the estimates or the inferences. No significant cluster effects (practice, therapist or teacher) were found, except for enablement, where a practice clustering effect was found, so only these results are presented allowing for clustering. Baseline characteristics were similar for all variables (table 2) except there were fewer women in the Alexander technique groups, probably a chance finding. Including sex in the models did not alter the estimates, so the results are presented unadjusted.

**Figure fig1:**
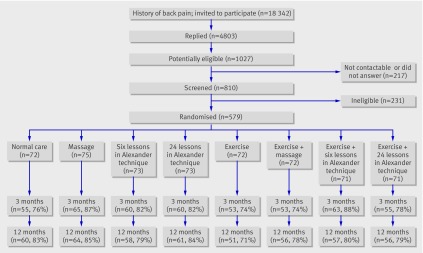
Flow of participants through trial

**Table 2 tbl2:** Comparison of groups at baseline according to two intervention factors (Alexander technique, exercise). Values are means (standard deviations) unless stated otherwise

Characteristic	Alexander technique factor					Exercise factor	
	Control	Massage	6 lessons	24 lessons		Control	Exercise
Roland disability score*	10.8 (4.8), n=144	11.3 (4.7), n=147	11 (5.3), n=144	10.7 (5.3), n=144		11.2 (5.2), n=293	10.7 (4.8), n=286
Age	46 (10), n=144	46 (10), n=147	45(11), n=144	45(11), n=143		45 (11), n=292	46 (10), n=286
No/total No women (%)	105/144 (73)	114/147 (78)	91/144 (63)	92/144 (64)		199/293 (68)	203/286 (71)
No/total No married (%)	79/133 (59)	84/142 (59)	88/139 (63)	79/140 (56)		163/282 (58)	167/272, (61)
Age on leaving full time education	18.0 (3.8), n=128	17.9 (3.9), n=140	17.8 (3.0), n=133	17.8 (3.5), n=133		17.9 (3.8), n=274	17.8 (3.3), n=260
No/total No employed (%)	96/131 (73)	108/143 (76)	104/137 (76)	102/140(73)		204/281 (73)	206/270 (76)
Von Korff overall†	4.7 (1.8), n=135	4.6 (1.8), n=140	4.8 (1.8), n=139	4.5 (1.8), n=139		4.7 (1.8), n=282	4.6 (1.8), n=271
Deyo troublesomeness‡	3.4 (0.6), n=135	3.4 (0.6), n=140	3.5 (0.7), n=140	3.3 (0.6), n=139		3.4 (0.7), n=282	3.3 (0.6), n=272
Median No of days (interquartile range) in pain in past four weeks§	24.5 (14-28), n=108	28 (14 to 28), n=116	28 (8 to 28), n=114	28 (13 to 28), n=115		28 (15 to 28), n=231	28 (14 to 28), n=222

*Number of activities affected by back pain.

†Severity of chronic pain scale.

‡Relative troublesomeness of pain in different body regions.

§As a result of an administrative error at baseline, not all patients had a questionnaire containing question for days in pain at baseline.

The trial population had predominantly chronic pain—on average 243 (SD 131) days of pain in the previous year. Seventy nine per cent reported 90 or more days of pain in the previous year.

### Outcomes at three months and one year

Little change occurred in Roland disability score or days in pain in the control group (table 3). Compared with the control group, significant reductions took place for all interventions for Roland disability score and days in pain at three months.

**Table 3 tbl3:** Outcomes at three months after randomisation. Values are mean differences compared with control group (95% confidence intervals) and P values, unless stated otherwise

Outcomes	Mean (SD) control (Alexander technique factor)*	Mean difference compared with control, P value			Mean (SD) control (exercise factor*)	Mean difference compared with control: exercise
		Massage	6 lessons in Alexander technique	24 lessons in Alexander technique		
**Primary outcomes**						
Roland disability score† (n=469)	9.34 (4.76)	-1.96 (-0.74 to 3.18), P=0.002	-1.71 (-2.95 to -0.47), P=0.007	-2.91 (-4.16 to 1.66), P<0.001	8.35 (4.75)	-0.90 (-1.76 to 0.04), P=0.04
Median (95% CI) No of days with back pain in past 4 weeks (n=405)‡	24 (21 to 27)	-13 (-18 to -8), P<0.001	-11 (-16 to -6), P<0.001	-16 (-21 to -11) P<0.001	17 (15 to 19)	-6 (-9 to -3), P<0.001
**Secondary outcomes**						
SF-36: quality of life physical§ (n=403)	54.9 (16.5)	2.57 (-2.20 to 7.34), P=0.290	4.39 (-0.40 to 9.19), P=0.072	7.5 (2.60 to 12.4), P=0.003	56.6 (16.5)	3.0 (-0.22 to 6.23), P=0.068
SF-36: quality of life mental§ (n=398)	62.5 (17.3)	-0.37 (-5.37 to 4.64), P=0.886	2.88 (-2.18 to 7.94), P=0.264	3.36 (-1.82 to 8.53), P=0.203	62.5 (17.2)	4.04 (0.65 to 7.43), P=0.020
Modified enablement scale¶ (n=386)	3.78 (1.15)	1.43 (1.10 to 1.76), P<0.001	1.45 (1.11 to 1.80), P<0.001	1.82 (1.47 to 2.16), P<0.001	4.80 (1.15)	0.41 (0.17 to 0.64), P=0.001
Von Korff overall** (n=412):	3.89 (1.71)	-0.13 (-0.60 to 0.35), P=0.597	-0.18 (-0.66 to 0.30), P=0.462	-0.47 (-0.96 to 0.02), P=0.061	3.83 (1.70)	-0.26 (-0.59 to 0.07), P=0.126
Von Korff disability††	3.27 (1.90)	0.00 (-0.51 to 0.52), P=0.993	0.00 (-0.52 to 0.52), P=0.990	-0.22 (-0.74 to 0.31), P=0.170	3.33 (1.90)	-0.25 (-0.61 to 0.11), P=0.170
Von Korff pain††	4.62 (1.85)	-0.41 (-0.91 to 0.09), P=0.110	-0.48 (-0.98 to 0.028), P=0.064	-0.75 (-1.26 to -0.24), P=0.004	4.39 (1.84)	-0.32 (-0.66 to 0.03), P=0.074
Deyo troublesomeness‡‡ (n=449)	3.09 (0.72)	-0.22 (-0.41 to -0.03), P=0.026	-0.20 (-0.40 to 0.01), P=0.039	-0.33 (-0.52 to -0.13), P=0.001	2.98 (0.72)	-0.11 (-0.24 to 0.02), P=0.103
Health transition§§ (n=433)	3.84(0.91)	-0.94 (-1.19 to -0.70), P<0.001	-0.81 (-1.06 to -0.56), P<0.001	-1.10 (-1.36 to -0.85), P<0.001	3.23 (0.91)	-0.22 (-0.39 to -0.05), P=0.013
Fear avoidance for physical activity (n=404)¶¶	14.2 (5.0)	-0.58 (-2.0 to 0.86), P=0.432	-0.80 (-2.25 to 0.64), P=0.276	-1.93 (-3.41 to -0.45), P=0.011	14.3 (5.0)	-2.70 (-3.68 to -1.72), P<0.001
Back health (n=407)***	3.35 (1.40)	1.56 (1.16 to 1.96), P<0.001	1.48 (1.08 to 1.89), P<0.001	1.84 (1.43 to 2.25), P<0.001	4.33 (1.40)	0.53 (0.26 to 0.80), P<0.001

*Effects in each factor are mutually controlled for other factor. Thus the control group for each factor are those that did not receive interventions for that factor. Interventions for each factor are expressed as estimated difference compared with control group with 95% confidence intervals. For example, control group had mean Roland score of 9.34, and massage groups had mean Roland score 1.96 lower than control group when adjusted for effect of exercise.

†Number of activities affected by back pain; 28=worst 0=best.

‡As a result of an administrative error at baseline, not all patients had a questionnaire containing question for days in pain at baseline, so model for days in pain does not include baseline values.

§0=worst, 100=best.

¶Mean of six items; 0=worst 6=best.

**Mean of six items; 10=worst 0=best.

††Mean of three items; 10=worst 0=best.

‡‡Mean of three items; 5=worst, 1=best.

§§Back pain changed; 7=vastly worsened, 4=no change, 1=completely recovered.

¶¶Sum of four items; 24=worst, 0=best.

***Mean of four items; 0=worst 6=best.

The effect of 24 lessons in the Alexander technique was greater at one year than at three months, with a 42% reduction in Roland disability score and an 86% reduction in days in pain compared with the control group (table 4). The effect of six lessons was maintained—a 17% reduction in Roland disability score and a 48% reduction in days in pain. Exercise still had a significant effect on Roland disability score (17% reduction) but not on days in pain. Massage no longer had an effect on Roland disability score but days in pain was reduced (by 33%). Twenty four lessons in the Alexander technique also had a significant effect on other outcomes; similar but smaller changes followed six lessons. Massage produced little change in other outcomes except perception of overall improvement in back pain (health transition), enablement, and overall satisfaction.

**Table 4 tbl4:** Outcomes at one year after randomisation: mean difference compared with control group (95% confidence intervals) unless specified otherwise

Outcomes	Mean (SD) control (Alexander technique factor*)	Mean difference compared with control, P value			Mean (SD) control (exercise factor*)	Mean difference compared with control: exercise
		Massage	6 lessons in Alexander technique	24 lessons in Alexander technique		
**Primary outcomes**						
Roland disability score (n=462)	8.07 (6.13)	-0.58 (-1.94 to 0.77), P=0.399	-1.40 (-2.77 to -0.03), P=0.045	-3.40 (-4.76 to -2.03), P<0.001	7.54 (6.25)	-1.29 (-2.25 to -0.34), P=0.008
Median (95% CI) No of days with back pain in past 4 weeks (n=435)†	21 (18 to 25)	-7 (-12 to -2), P=0.004	-10 (-15 to -5), P<0.001	-18 (-23 to -13), P<0.001	13 (11 to 15)	-2 (-5 to 1), P=0.233
**Secondary outcomes**						
SF-36: quality of life physical (n=403)	56.4 (18.5)	1.7 (-4.0 to 7.4), P=0.553	6.0 (0.30 to 11.6), P=0.039	11.3 (5.7 to 16.9), P<0.001	59.5 (18.5)	1.9 (-1.97 to 5.79), P=0.333
SF-36: quality of life mental (n=341)	65.2 (17.4)	-0.1 (-5.5 to 5.2), P=0.956	2.0 (-3.4 to 7.5), P=0.460	4.0 (-1.4 to 9.3), P=0.145	66.5 (17.3)	0.9 (-2.8 to 4.6), P=0.636
Modified enablement scale (n=366)	3.80 (1.20)	1.29 (0.93 to 1.64), P<0.001	1.31 (0.95 to 1.67), P<0.001	1.80 (1.44 to 2.16), P<0.001	4.69 (1.19)	0.50 (0.24 to 0.76), P<0.001
Von Korff overall (n=412):	3.96 (2.32)	-0.02 (-0.64 to 0.59), P=0.939	-0.60 (-1.22 to 0.007), P=0.053	-1.15 (-1.75 to -0.55), P<0.001	3.83 (2.36)	-0.59 (-1.01 to -0.17), P=0.006
Von Korff disability	3.34 (2.24)	0.03 (-0.63 to 0.68), P=0.938	-0.57 (-1.23 to 0.08), P=0.085	-0.95 (-1.60 to -0.30), P=0.004	3.22 (2.23)	-0.59 (-1.04 to -0.14), P=0.011
Von Korff pain	4.54 (2.19)	-0.01 (-0.65 to 0.63), P=0.981	-0.58 (-1.22 to 0.06), P=0.075	-1.30 (-1.93 to -0.67), P<0.001	4.40 (2.18)	-0.59 (-1.04 to -0.14), P=0.011
Back health transition (n=430)	3.67 (1.14)	-0.63 (-0.93 to -0.32), P<0.001	-0.55 (-0.86 to -0.24), P<0.001	-0.97 (-0.75 to -0.31), P<0.001	3.38 (2.83)	-0.53 (-0.75 to -0.31), P<0.001
Deyo troublesomeness (n=462)	2.94 (0.75)	0.05 (-0.16 to 0.26), P=0.627	-0.16 (-0.37 to 0.05), P=0.132	-0.34 (-0.55 to -0.12), P=0.002	2.94 (0.85)	-0.16 (-0.31 to -0.01), P=0.036
Fear avoidance for physical activity (n=350)	13.6 (5.3)	-0.23 (-1.86 to 1.39), P=0.777	-1.41 (-3.03 to 0.21), P=0.088	-2.28 (-3.90 to -0.67), P=0.006	13.2 (5.3)	-1.87 (-2.99 to -0.75), P=0.001
Back health (n=362)	3.44 (1.45)	1.13 (0.69 to 1.56), P<0.001	1.26 (0.82 to 1.71), P<0.001	1.82 (1.38 to 2.25), P<0.001	4.15 (1.45)	0.74 (0.44 to 1.04), (P<0.001
Satisfaction with overall management (n=319)	3.17 (1.04)	0.47 (0.11 to 0.82), P=0.01	0.58 (0.22 to 0.93), P=0.001	0.70 (0.35 to 1.04), P<0.001	3.45 (1.21)	0.47 (0.22 to 0.71), P=0.001

See table 3 for definitions of scores.

Cronbach’s a for scales: Deyo troublesomeness 0.87, Von korff 0.95, fear avoidance 0.80, enablement 0.96, back health 0.96.

*Effects in each factor are mutually controlled for other factor. Thus the control group for each factor are those that did not receive interventions for that factor. Interventions for each factor are expressed as estimated difference compared with control group, with 95% confidence intervals.

†As a result of an administrative error at baseline, not all patients had a questionnaire containing question for days in pain at baseline, so model for days in pain does not include baseline values.

### Adherence

Good adherence was defined by the trial management group as attending five out of six massage sessions, five out of six lessons in the group randomised to six lessons in the Alexander technique, and 20 out of 24 lessons in the group randomised to 24 lessons. Good adherence was achieved by 91% (108/119), 94% (106/113), and 81% (95/117), respectively. For exercise prescription—when repeated attendance was not necessary to increase physical activity—the management group judged that adequate adherence was seeing the general practitioner once (for the prescription) and the nurse at least once (for behavioural counselling and reinforcement); this was achieved by 76% (211/278) of patients. No meaningful change occurred in the results when only those patients with good adherence were selected.

### Individual groups

The effect of exercise combined with 24 Alexander technique lessons on Roland disability score and other outcomes was similar to the effect of 24 lessons alone (table 5). The effect of six lessons followed by exercise prescription on Roland disability score and most other outcomes was almost as good (72% as effective) as 24 lessons.

**Table 5 tbl5:** Individual groups one year after randomisation

Outcomes	Mean (SD) control (no exercise)	Massage	6 lessons in Alexander technique	24 lessons in Alexander technique	Exercise	Exercise+massage	Exercise+6 lessons in Alexander technique	Exercise+24 lessons in Alexander technique
**Primary outcomes**								
Roland disability score	9.23 (5.3)	-0.45 (-2.3 to 1.39), P=0.629)	-1.44 (-3.34 to 0.45), P=0.135	-4.14 (-6.01 to -2.27), P<0.001	-1.65 (-3.62 to 0.31), P=0.099	-2.37 (-4.28 to -0.47), P=0.015	-2.98 (-4.88 to -1.07), P=0.002	-4.22 (-6.13 to -2.31), P=0.002
Median (95% CI) No of days with back pain in past 4 weeks	23 (14 to 28)	-8 (-20 to 4), P=0.178	-13 (-25 to -1), P=0.034	-20 (-28 to -8), P=0.001	-11 (-23 to -1), P=0.084	-11 (-23 to -1), P=0.080	-13 (-25 to -1), P=0.031	-20 (-28 to -8), P=0.001
**Secondary outcomes**								
Modified enablement scale	3.38 (1.20)	1.31 (0.88 to 1.75, P<0.001	1.53 (0.97 to 2.08), P<0.001	2.19 (1.69 to 2.69), P<0.001	0.89 (0.31 to 1.48), P<0.001	2.10 (1.60 to 2.59), P<0.001	1.91 (1.46 to 2.36), P<0.001	2.24 (1.78 to 2.69), P<0.001
SF-36: quality of life physical	56.1 (18.6)	-1.45 (-9.04 to 6.15), P=0.708	2.04 (-5.58 to 9.67), P=0.599	11.83 (4.24 to 19.4), P=0.002	-2.08 (-10.6 to 6.40), P=0.629	3.63 (-4.13 to 11.4), P=0.358	8.53 (0.86 to 16.20), P=0.029	9.43 (1.88 to 16.97), P=0.015
SF-36: quality of life mental	64.8 (17.5)	-2.11 (-9.37 to 5.16), P=0.569	4.10 (-3.27 to 11.5), P=0.274	3.74 (-3.56 to 11.0), P=0.314	0.72 (-7.38 to 8.81), P=0.862	2.73 (-4.69 to 10.1), P=0.470	0.64 (-6.79 to 8.07), P=0.866	4.99 (-2.31 to 12.3), P=0.180
Von Korff overall:	4.19 (2.11)	0.31 (-0.52 to 1.14), P=0.464	-0.30 (-1.13 to 0.53), P=0.483	-1.10 (-1.92 to -0.28), P=0.009	-0.19 (-1.09 to 0.72), P=0.684	-0.61 (-1.46 to 0.23), P=0.154	-1.17 (-2.01 to -0.33), P=0.007	-1.44 (-2.26 to -0.61), P=0.001
Von Korff disability	3.32 (2.25)	0.46(-0.43 to 1.35), P=0.313	-0.08 (-0.97 to 0.81), P=0.854	-0.78 (-1.66 to 0.09), P=0.079	0.05 (-0.92 to 1.02), P=0.924	-0.45 (-1.36 to 0.45), P=0.324	-1.11 (-2.02 to -0.22), P=0.016	-1.14 (-2.03 to -0.26), P=0.011
Von Korff pain	4.74 (2.20)	0.29 (-0.58 to 1.16), P=0.510	-0.44 (-1.31 to 0.44), P=0.327	-1.32 (-2.18 to -0.26), P=0.003	-0.31 (-1.26 to 0.63), P=0.516	-0.66 (-1.55 to 0.22), P=0.140	-1.08 (-1.96 to -0.20), P=0.017	-1.63 (-2.49 to -0.76), P<0.001
Back health transition	3.93 (1.15)	-0.53 (-0.95 to -0.12), P=0.012	-0.55 (-0.98 to -0.12), P=0.013	-1.11 (-1.54 to -0.68), P<0.001	-0.55 (-1.0 to -0.1), P=0.017	-1.29 (-1.72 to -0.86), P<0.001	-1.10 (-1.52 to -0.67), P<0.001	-1.38 (-1.80 to -0.95), P<0.001
Deyo troublesomeness	3.05 (0.80)	0.04 (-0.25 to 0.33), P=0.771	-0.13 (-0.42 to 0.16), P=0.380	-0.46 (-0.76 to -0.17), P=0.002	-0.21 (-0.52 to 0.09), P=0.175	-0.15 (-0.45 to 0.15), P=0.324	-0.40 (-0.70 to -0.11), P=0.007	-0.42 (-0.72 to -0.12), P=0.006
Fear avoidance for physical activity	14.5 (5.35)	-0.88 (-3.05 to 1.29), P=0.427	-0.92 (-3.11 to 1.26), P=0.405	-3.00 (-5.19 to -0.80), P=0.008	-2.41 (-4.84 to 0.02), P=0.052	-1.84 (-4.07 to 0.38), P=0.104	-4.23 (-6.43 to -2.03), P<0.001	-3.90 (-6.06 to -1.74), P<0.001
Back health	3.07 (1.46)	0.94 (0.35 to 1.53), P=0.002	1.22 (0.63 to 1.81), P<0.001	2.01 (1.43 to 2.60), P<0.001	0.72 (0.06 to 1.39), P=0.033	2.05 (1.45 to 2.64), P<0.001	2.03 (1.43 to 2.63), P<0.001	2.34 (1.75 to 2.93), P<0.001

See table 3 for definitions of scores.

### Adverse events

One patient mentioned that their back pain had been made considerably worse by massage. No adverse events were reported for exercise or Alexander technique lessons.

## Discussion

A series of 24 lessons in the Alexander technique taught by registered teachers provides long term benefits for patients with chronic or recurrent low back pain. Both six lessons in the Alexander technique and general practitioner prescription for aerobic exercise with structured behavioural counselling by a practice nurse were helpful in the long term; classic massage provided short term benefit. Six lessons in the Alexander technique followed by exercise prescription was almost as effective as 24 lessons.

Most patients we contacted were not eligible. The majority of the eligible patients who responded to an invitation to participate in the trial were randomised so the results should apply to most patients with chronic or recurrent back pain. The long previous duration of pain (79% had pain for >90 days) and the little change in pain and function in the control group after one year (still had significant limitation in activity and pain on most days after one year) suggest that we selected a predominantly chronic, severely affected, and currently ineffectively managed population. All had attended primary care with back pain in the past—that is, the sample was a clinically relevant population. Since patients were required to be able to walk, we excluded those most severely disabled by pain.

Adherence was good for both six and 24 lessons in the Alexander technique, and for massage compared with adherence in other back pain intervention trials,^7^ possibly as a result of the perceived symptomatic benefit. As this was a large pragmatic, multipractice, multiteacher, multitherapist study, the results are unlikely to be due to the good work of a small number of enthusiasts.

The consistent pattern of outcomes at three months and one year and number of highly significant results suggest that a type I error (chance) was unlikely. The study was powered to detect a reduction of 1.5 to 2.5 activities affected by back pain. Although the study was underpowered to assess significant interactions (none was found) the results suggest that the effect of exercise and 24 Alexander technique lessons combined is less than the sum of the two individual effects. We found no evidence of confounding or bias from losses to follow-up.

The Roland disability scale is one of the best validated self report measures for assessing the impact of back pain.^21^ ^22^ The effect of intervention on our other primary outcome, reported days in pain, is unlikely to be explained by recall bias owing to the large effect size and short period of recall. Recall over such periods is likely to be valid: pain or discomfort for both short recall periods (2-4 weeks) and longer recall periods in a variety of conditions compare favourably with diaries completed prospectively.^32^ ^33^ ^34^ Any non-differential measurement error owing to the use of reported days in pain is likely to underestimate true differences between groups.

### Interventions

#### Alexander technique lessons

The previous trial for back pain was smaller and involved one teacher.^16^ Our study shows enduring benefits from lessons delivered by many different teachers. That six sessions of massage were much less effective at one year than at three months whereas six lessons in the Alexander technique retained effectiveness at one year shows that the long term benefit of Alexander technique lessons is unlikely to result from non-specific placebo effects of attention and touch.

#### Massage

Massage is helpful in the short term, which supports tentative conclusions from previous research.^17^ ^19^ Benefit in the longer term is probably less, which is supported by previous comparison with a self care booklet,^35^ although this trial did find benefit compared with acupuncture. Acupressure may possibly be more effective than the classic massage we used.^17^

#### Exercise

Prescription from a general practitioner for unsupervised home based aerobic exercise (predominantly walking) with follow-up structured counselling, based on the theory of planned behaviour,^36^ and using behavioural principles, provided modest but useful benefits from a relatively brief intervention. Comparison with the United Kingdom back pain exercise and manipulation trial suggests the benefits are similar to a supervised exercise scheme in the short term, and potentially greater in the long term, since the effect of supervised schemes in that trial was no longer apparent by 12 months.^7^ Six lessons on the Alexander technique followed by prescription for exercise provided nearly as much benefit as 24 lessons on the Alexander technique.

### Other interventions

A recent study of acupressure in a Chinese orthopaedic clinic^37^ and single practitioner trial of yoga suggest substantial benefit for back pain,^38^ but trials were small (<130 participants) with six months of follow-up. Systematic reviews of manipulation suggest limited benefit,^10^ and the United Kingdom back pain exercise and manipulation trial showed moderate benefits from manipulation combined with supervised exercise at one year (1.3 reduction in Roland disability score). A systematic review suggested that strengthening and stabilising exercises are likely to have moderate benefit^4^; the more pronounced effects in a recent trial^39^ require confirmation as the follow-up rate was poor (<60%). The finding of possible benefit of acupuncture for quality of life at 24 months but not 12 months^40^ requires confirmation, given the negative findings for pain and disability^40^ and the negative long term findings reported in the Cochrane review.^41^ The magnitude of benefit we found in the current study—of 3 points on the Roland disability score—is likely to be important for patients: an improvement of 3 points on the score means that patients have three fewer activities or functions limited by back pain (such as being able to get out of the house less often, walking more slowly than usual, not doing usual jobs around the house). This benefit can be provided by 24 lessons in the Alexander technique, or six lessons combined with exercise prescription.

**What is already known on this topic**

Combined manipulation and physiotherapy-supervised strengthening exercises helps functioning moderately (1-2 activities no longer limited by back pain)
Preliminary evidence suggests that massage and lessons in the Alexander technique might help in the short term

**What this study adds**

Six sessions of massage, prescription for exercise and nurse counselling, six lessons in the Alexander technique, and 24 lessons helped with back pain and functioning at three months
Lessons in the Alexander technique still had a beneficial effect on pain and functioning after 12 months
Six lessons in the Alexander technique followed by exercise prescription are nearly as effective as 24 lessons

## Notes

We thank the patients, practices, general practitioners, practice nurses, teachers, and therapists for their time and effort; Carolyn Nicholls, who helped develop the Alexander technique teachers’ record forms and organised the pilot of the trial; Andy Fagg, who helped develop the massage intervention; and the trial steering committee (chairman Martin Underwood) for their help throughout the trial.

Contributors: PL and GL had the original idea for this protocol; DS and FO had been working on a parallel protocol. The protocol was developed by all the authors. PL led the grant application in conjunction with DS, GL, and PS (principal investigators). FW coordinated the trial on a day to day basis supervised by PL and helped by JB and KM, and managed the Southampton site. KM managed the database, supervised by PS. ME and AB managed the Bristol site on a day to day basis, supervised by DS. KB and FO coordinated the development of the Alexander technique intervention and monitoring. LY coordinated the development of the exercise prescription intervention and monitoring. All authors contributed to regular meetings on trial management. PL and PS did the analysis, which was discussed by all authors. PL wrote the paper, and all authors contributed to revisions of the paper. PL is guarantor for the paper.

Ethical approval: South west multicentre research ethics committee (reference 01/6/54).

Provenance and peer review: Not commissioned; externally peer reviewed.

## Notes

**Cite this as:** *BMJ* 2008;337:a884
